# Hypothermia as an adjunctive therapy to percutaneous intervention after ST-elevation myocardial infarction—Effects on regional myocardial contractility

**DOI:** 10.1016/j.jocmr.2025.101850

**Published:** 2025-02-13

**Authors:** Lucas de Mello Queiroz, Rafael Almeida Fonseca, Luis Augusto Palma Dallan, Thatiane Facholi Polastri, Ludhmila Abrahao Hajjar, Jose Carlos Nicolau, Roberto Kalil Filho, Karl B. Kern, Sergio Timerman, Carlos E. Rochitte

**Affiliations:** a University of Sao Paulo Medical School, Sao Paulo, SP, Brazil; b Heart Institute, InCor, University of Sao Paulo Medical School, Sao Paulo, Sao Paulo, SP, Brazil; c Department of Cardiovascular Medicine, Harrington Heart and Vascular Institute, University Hospitals Cleveland Medical Center, Cleveland, Ohio, USA; d Division of Cardiovascular Medicine, Sarver Heart Center, University of Arizona, Tucson, Arizona, USA

**Keywords:** Cardiac magnetic resonance, Myocardial strain, Therapeutic hypothermia, ST-elevation myocardial infarction, Percutaneous coronary intervention

## Abstract

**Background:**

The effects of endovascular therapeutic hypothermia (ETH) in ST-elevation myocardial infarction (STEMI) regional contractility are unknown, and its impact on segmental contractility has still not been evaluated. We sought to evaluate segmental myocardial strain after ETH adjuvant to percutaneous coronary intervention (PCI) in STEMI.

**Methods:**

We included patients who underwent 1.5T cardiovascular magnetic resonance exams 5 and 30 days after acute anterior or inferior STEMI in a previous randomized trial. Left ventricle (LV) strain was evaluated on infarcted, adjacent, and remote myocardium. Segmental circumferential (CS) and radial strains (RS) were measured using feature-tracking imaging. Repeated measures of analysis of variance were used for comparisons within time and treatment.

**Results:**

Forty patients were divided into hypothermia (ETH, n = 29) and control (n = 11) groups, with 5210 LV segments. In ETH infarcted areas, RS (11.2 ± 16 vs 14.8 ± 15.2, p = 0.001) and CS (−5.4 ± 11.1 vs −8 ± 11.1, p = 0.001) showed recovery from 5–30 days compared to controls (11.4 ± 14 vs 13.1 ± 1 6.8, p = 0.09; −6.5 ± 10.6 vs −6.4 ± 12.5, p = 0.94). In control remote areas, RS (28 ± 18 vs 31.7 ± 18.5, p = 0.001) and CS (−15.5 ± 10.7 vs −17.1 ± 9, p = 0.001) improved from 5–30 days compared to ETH (28.6 ± 18.6 vs 29 ± 20, p = 0.44; −15.2 ± 10.4 vs −15.3 ± 10.6, p = 0.82). Transmural infarcted areas in ETH improved RS (11.8 ± 13.2 vs 8.17 ± 14.7, p = 0.001) and CS (−6.1 ± 10.9 vs.−3.1 ± 11.3, p = 0.001) compared to controls, with better contractility at 30 days.

**Conclusion:**

In anterior or inferior STEMI patients, ETH adjuvant to PCI is associated with significant improvement in RS and CS of infarcted areas, including transmural segments, but not in remote area. This might further increase our pathophysiological knowledge on early LV remodeling and ultimately suggest potential clinical value.

**Availability of data and materials:**

The datasets used and/or analyzed during the current study are available from the corresponding author upon reasonable request.

## Graphical abstract

**Figure:**
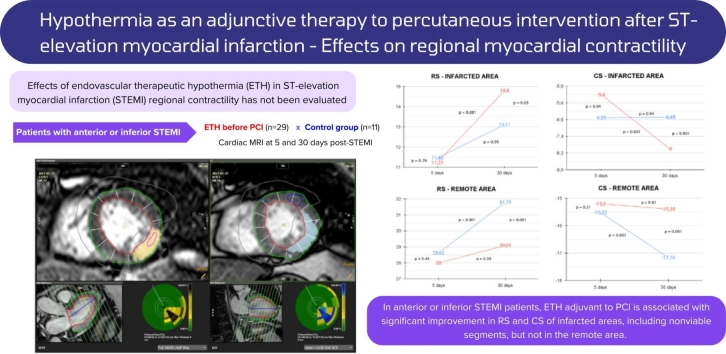

## 1 Introduction

Cardiovascular diseases (acute myocardial infarction in a special way) remain the main cause of death in Brazil and controversies persist involving its optimal management, despite recent advances [1]. Endovascular therapeutic hypothermia (ETH) revealed a positive reduction of postischemic reperfusion damage after cardiac arrest [2], [3], [4], [5]. However, current studies are still controversial regarding the role of ETH in ST-elevation myocardial infarction (STEMI) [6]. Randomized clinical trials, including COOL-MI [7], ICE-IT [8], CHILL-MI [9], VELOCITY [10], COOL AMI EU PILOT [11], and the COOL-MI InCor Trial [12], [13], demonstrated that ETH before percutaneous coronary intervention (PCI) is feasible and safe. These studies have shown that ETH implementation can be performed without delay in coronary reperfusion, but the role of the intervention on infarction size (IS), left ventricular ejection fraction (LVEF), and other LV parameters is poorly understood.

STEMI is known to impact its adjacent portions and the remote walls of the heart [14], [15]. In the COOL-MI InCor Trial [16], a higher incidence of arrhythmias was found in the hypothermia group, suggesting a possible ETH influence on myocardial contractility, which supports the relevance of further studies for elucidation. Additionally, studies on the impact of hypothermia on non-infarcted LV contractility are not available.

Assessment of contractile dysfunction severity in patients with STEMI allows evaluation of prognosis and may help tailor therapeutic regimens [16], [17]. Most of the existing ETH studies analyze LVEF changes [7], [8], [9], [10], [11], [12]. Nevertheless, LVEF is a global measurement with low sensitivity for detecting regional myocardial disorders. Moreover, regional contractility is evaluated in the clinical scenario using visual and qualitative analysis, which carries a high interobserver variability.

Myocardial strain analysis by cardiovascular magnetic resonance feature tracking (CMR-FT) is an accurate, efficient, and highly reproducible technique for the early detection of segmental LV dysfunctions [18], with good reproducibility in various pathologies and between genders [19]. CMR-FT robustness [20] has been validated for global and segmental strain analysis after acute myocardial infarction, despite the increase in post processing time. Therefore, strain data weight in myocardial contractility evaluation [21], [22], as it is an objective and quantitative measure, which also provides valuable prognostic information in the short- and long-term post-STEMI [23], [24], [25], [26], [27].

We sought to investigate whether ETH as adjunctive therapy to primary PCI affects myocardial strains in infarcted, adjacent, and remote segments, in patients with anterior or inferior STEMI.

## 2 Methods

This study was a retrospective analysis of CMR exams available in the image database of the COOL-MI InCor Trial, a single-center randomized controlled trial, performed at InCor, Heart Institute, Clinical Hospital, University of São Paulo, São Paulo, Brazil.

### 2.1 Patient population

We included patients ≥18 years of age admitted to the emergency department with up to 6 h of symptom onset, presenting with an anterior or inferior STEMI with persistent ST-segment elevation of >0.2 mV in two contiguous leads at arrival at the catheterization laboratory. Patients treated with ETH as adjunctive therapy to PCI were selected for analysis in the hypothermia group; patients treated only with PCI were enrolled in the control group. All patients submitted to the CMR exam with the protocol described below at 5 and 30 days of follow-up were included in the present analysis.

The exclusion criteria were resuscitated cardiac arrest, previous myocardial infarction, PCI, or coronary artery bypass grafting, Killip class II–IV at presentation, atrial fibrillation, end-stage kidney disease or hepatic failure, recent ischemic or hemorrhagic stroke (<3 months), coagulopathy, and pregnancy.

The study was performed in accordance with the Declaration of Helsinki and the local ethics committee approved the study protocol. Treatment provided before cooling, the cooling protocol, and the randomization methods were described in detail previously [12], [13].

### 2.2 Cardiovascular magnetic resonance imaging acquisition

All studies were performed with 1.5T CMR scanners (Achieva, Philips Healthcare, Best, Netherlands or Vantage Titan, Toshiba Medical Systems, Tokyo, Japan), with the same protocol, which included LV short- and long-axis cine images (steady-state free precession [SSFP] sequences) and late gadolinium enhancement (LGE, using standard segmented inversion-recovery prepped gradient-echo sequence) in the same slice positions as cine images. This protocol allowed an accurate comparison of cardiac function and regional myocardial structure.

The parameters for cine-MR (SSFP) images were repetition time (TR) of 3.5 ms, echo time (TE) of 1.5 ms, flip angle 60°, receipt bandwidth of ±125 kHz, field of view (FOV) of 35 × 35 cm, 256 × 148 matrix, temporal resolution 35 ms, 8.0 mm slice thickness, with 2 mm gap between the slices.

For edema evaluation, T2-weighted images were obtained using a triple inversion-recovery pulse fast spin-echo (dark blood), and long TEs (>70 ms, 100 ms ideally), with breath-hold, in short-axis view of the left ventricle (LV). In this sequence, volumetric body coil acquisition was used to guarantee volume homogeneity and avoid signal falls, as occurs with surface coils. The parameters were TR of 2 RR interval, TE from 80–120 ms, echo train length 24, inversion time (TI) of 140 ms, slice thickness of 10 mm, a gap of 2 mm, FOV of 34 × 38 cm, and matrix 256 × 256. We also acquired cine SSFP after contrast as an additional measurement of myocardial edema and area at risk (AAR).

Following the acquisition of spin-echo images, a dose of gadolinium-based contrast (0.2 mmol/kg) was injected. The LGE technique was used for detecting myocardial infarction. Inversion-recovery prepared gradient-echo was acquired 10–20 min after the contrast administration (Dotarem, Guerbet, Villepinte, France) dosed at 0.2 mmol/kg body weight with the following parameters: TR 7.1 ms, TE 3.1 ms, flip angle 20°, cardiac phase 1, 16–32 views per segment (depending on heart rate), matrix 256 × 192, slice thickness 8 mm, gap between slices of 2 mm and FOV of 32–8 cm, IT from 150–250 ms (adjusted to null normal myocardium signal), receiver bandwidth of 31.25 kHz, number of excitations 2, acquisition every-other heartbeat (2 RR acquisition).

### 2.3 Image analysis

All CMR exams were analyzed using a dedicated CMR-FT software (CVi42 5.9.4, Circle Cardiovascular Imaging Inc., Calgary, Alberta, Canada), which allows strain parameter analysis based on the user definition of myocardial borders in standard cine SSFP images.

All cine sequences throughout the cardiac cycle were submitted to an automated tracking algorithm. Selected frames were submitted to semiautomatic delineation of LV endo- and epicardial borders. Tracking performance was visually and manually reviewed in all slices to ensure accurate tracking. In the case of inadequate automated border tracking, myocardial contours were manually readjusted in all datasets. Papillary muscles and myocardial trabeculations were included in the ventricular cavity. The mitral valve plane and apex were drawn on long-axis sections at end diastole. End-diastolic and end-systolic phases were used to determine LVEF, LV end-diastolic volume (LVEDV), LV end-systolic volume, and LV end-diastolic mass (LV mass).

**Fig. 1:**
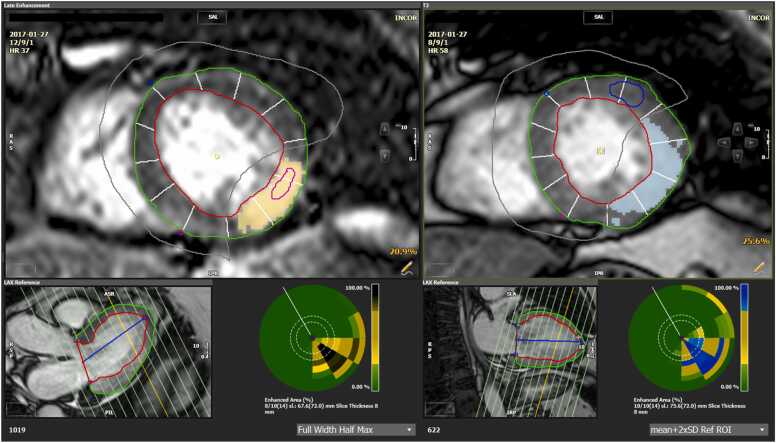
Short-axis LV view illustrating myocardial segmentation into infarcted, adjacent, and remote regions. The LV short-axis view is divided into 12 circumferential segments, each categorized according to its proximity to the infarcted myocardium. Infarcted segments are identified by late gadolinium enhancement (LGE) as hyperintense areas (yellow area on the left CMR). Adjacent segments are those contiguous with the infarcted segments but without direct LGE enhancement. Remote segments are regions without LGE, located on the LV wall opposite to the infarcted area The blue-highlighted region represents the area of myocardial edema, as visualized in the T2-weighted imaging sequence. *LV* left ventricle, *CMR* cardiovascular magnetic resonance, *ASR* anterior superior right, *SLA* superior lateral anterior, *RAS* right anterior superior, *HR* heart rate, *LAX* long-axis, *IPR* inferior posterior right, *RPS* right posterior superior, *PIL* posterior inferior lateral, *ROI* region of interest, *SAL* superior anterior lateral, *RSP* right superior posterior.

Strain parameters were evaluated in the short-axis view divided into 12 circumferential segments that were categorized as infarcted, adjacent, and remote to the infarcted myocardium (Fig. 1). Infarcted areas were identified and quantified as an enhancement on LGE images, using the full width of half-max technique, including areas of microvascular obstruction. Adjacent segments were defined as those segments without infarction by LGE but immediately connected to both sides of infarcted segments in the LV short-axis view. Remote segments were defined as segments without infarction that were not adjacent to the infarcted segments and were thus surrounded by segments without infarction. The remote segments were usually located on the opposite left ventricular wall.

Infarcted areas were divided into subendocardial and transmural, considering the probability of contractility recovery after revascularization [27]. Subendocardial segments were those with LGE involving less than 50% of their area, and transmural segments had LGE involving more than 50% of their area.

Longitudinal strain (LS) parameters were derived on long-axis cines, as a global measure. Circumferential strain (CS) and radial strain (RS) were calculated in a two-dimensional mode, globally and for each of the 12 segments in each segment category (infarcted, adjacent, and remote) for each slice, on short-axis cines. All slices with infarction and the first next slice were included. The first slice without infarction was included to consider its adjacent areas. The cross-matching correlation between LGE images and cine-MR images was performed by matching the slice location of the images and confirming the matching anatomical position by visualizing side-by-side LGE and cine-MR images, through matching of the papillary muscles.

The AAR size was assessed by an evaluation of tissue edema in SSFP cine images acquired after LGE at 5 days, considering a threshold signal intensity >2 standard deviation (SD) above the mean of a normal region included in a remote segment. Myocardial salvage (MS) is considered a measure of reperfusion strategy efficacy, as it has important prognostic implications [28]. AAR, MS, and myocardial salvage index (MSI) were calculated as follows:

a. AAR = edema volume/LV mass volume
b. MS = AAR − infarct size
c. MSI = (AAR − infarct size)/AAR

### 2.4 Statistical analysis

Continuous variables were expressed as mean ± SD. Categorical variables were expressed as absolute numbers and percentages. Normality was confirmed using the Shapiro-Wilk test and skewness test, as appropriate. All statistical analyses were performed with STATA 13.1 software (StataCorp, College Station, Texas, USA). All p-values were two-tailed and values <0.05 were considered statistically significant. For continuous variables, t-test (Gaussian distribution) and Mann-Whitney U tests (non-Gaussian distribution) were used to compare the two treatment groups. Repeated measures of analysis of variance were used to compare strain parameters with time and treatment, considering influences among wall segments within each patient. In this approach, we used a method that corrects the lack of independence between segments, by applying a correction to the degrees of freedom of the F test for each segment. T-test was only used to measure CMR morphofunctional parameters and clinical characteristics of the study population, and was not used in any case to analyze segmental strain parameters.

The intra and interobserver variability for strain calculation were evaluated in a random sample of eight individuals (10%) selected from each group. To determine intraobserver variability, the primary observer reanalyzed the selected exams 12 months after the first analysis, without reviewing previous measurements. To determine interobserver variability, a second observer analyzed the same data, blind to the measurements of the first observer. Intraclass correlation coefficient (ICC) estimates and their 95% confidence intervals (CI) were calculated using STATA 13.1 based on an absolute-agreement, two-way mixed-effects model.

## 3 Results

### 3.1 Patient characteristics

**Fig. 2:**
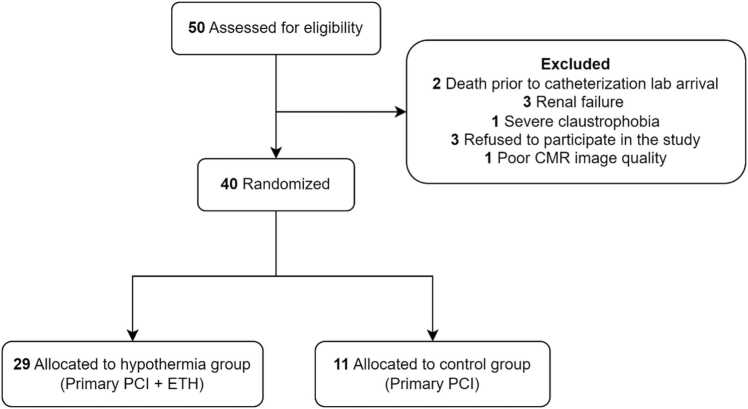
Flowchart describing patient randomization and exclusion reasons. *CMR* cardiac magnetic resonance, *PCI* percutaneous coronary intervention, *ETH* endovascular therapeutic hypothermia.

The clinical characteristics of the population are shown in Table 1. Fifty patients were included in the study. Two (4%) were excluded due to death before catheterization lab arrival. Three (6%) could not undergo CMR owing to renal failure, and one (2%) could not because of severe claustrophobia. Three (6%) refused to participate in the study. One remaining patient (2%) was excluded because images were inadequate for tissue-tracking analysis due to severe cardiac and respiratory motion artifacts. Therefore, 40 patients were selected and divided into hypothermia (primary PCI + ETH, n = 29) and control (primary PCI, n = 11) groups, with 5210 segments in total (3746 in the hypothermia group and 1646 in the control group). Fig. 2 describes population randomization and the reasons for exclusion.

**Table 1:** Clinical characteristics of study population.

	Control (n = 11)	Hypothermia (n = 29)	p-value
Average age (y)	53.54±10.49	58.24±8.46	0.15
Sex (male)	9 (82%)	23 (79%)	0.86
Weight (kg)	85.72±23.4	78.72±14.59	0.26
BMI	29.29±7.28	28.28±6.49	0.67
Hypertension	6 (55%)	17 (59%)	0.82
Diabetes	3 (27%)	11 (38%)	0.53
Dyslipidemia	4 (36%)	23 (79%)	0.01
Heart failure (EF <40%)	0 (0%)	2 (7%)	0.37
Renal disease	0 (0%)	1 (3%)	0.53
Family history of atherosclerosis	5 (45%)	7 (24%)	0.19
Smoking			
Current	5 (45%)	13 (45%)	0.97
Previous	6 (55%)	18 (62%)	0.66
MI wall			0.23
Anterior wall MI	3 (27%)	14 (48%)	
Inferior wall MI	8 (73%)	15 (52%)	
Infarct culprit artery			0.47
LAD	3 (30%)	15 (52%)	
RCA	3 (40%)	9 (31%)	
LCx	3 (30%)	5 (17%)	
Atrial fibrillation	2 (18%)	15 (52%)	0.055
Malign arrhythmia (VF or VT)	1 (9%)	7 (24%)	0.29
Previous PCI	0 (0%)	3 (10%)	0.27
GRACE score			0.36
≤108	6 (54%)	11 (38%)	
109–140	3 (27%)	13 (44%)	
>140	2 (18%)	5 (17%)	
Dorsal wall associated MI	4 (36%)	5 (17%)	0.20
Myocarditis	1 (9%)	0 (0%)	0.10
Troponin			
Admission	9.00±16.81	10.67±17.57	0.80
After 8 h	37.88±20.9	39.43±19.01	0.82
After 16 h	49.11±2.66	39.69±17.99	0.13
After 24 h	50.00±0	38.18±18.5	0.10
After 48 h	38.80±18.3	33;46±18.40	0.51
Time of chest pain (min)	266±91	291±86	0.41
Pain-to-balloon time	335±87	364±94	0.38
Door-to-balloon time	69±20	72±20	0.66
Presence of microvascular obstruction (no-reflow)	10 (91%)	23 (79%)	0.65
Number of stents (mean)	1.54±0.82	1.37±0.67	0.52
PCI myocardial localization			0.48
Basal	6 (54%)	21 (72%)	
Mid-cavity	4 (36%)	5 (17%)	
Apical	1 (9%)	3 (10%)	
Thrombectomy	0 (0%)	0 (0%)	
Residual stenosis			0.018
Absent	9 (82%)	29 (100%)	
Present	2 (18%)	0 (0%)	
TIMI flow pre-PCI			0.81
I	4 (36%)	11 (38%)	
II	2 (18%)	2 (7%)	
III	5 (45%)	16 (55%)	
Number of stents			0.12
0 stents	0 (0%)	1 (3%)	
1 stents	7 (63%)	18 (62%)	
2 stents	2 (18%)	8 (28%)	
3 stents	2 (18%)	2 (7%)	
Multivessel PCI			0.29
Absent	9 (82%)	27 (93%)	
1 additional PCI	2 (18%)	10 (36%)	
Staged PCI			0.29
Absent	9 (82%)	18 (64%)	
1 additional PCI	2 (18%)	10 (36%)	
Degree of new stenosis	60±14.14	57±18.88	0.84
Local of new stenosis			0.66
Basal	2 (18%)	5 (17%)	
Mid-cavity	0 (0%)	4 (14%)	
Apical	0 (0%)	1 (3%)	
Use of GP IIb/IIIa inhibitors	5 (50%)	4 (14%)	0.019
Medications post-MI			
Beta-blockers	7 (64%)	12 (41%)	0.21
ACE/ARB/ARNI	10 (91%)	26 (90%)	0.91
MRAs	1 (9%	7 (49%)	0.29

Data are expressed as the mean ± SD for numeric variables and as absolute values and percentages for categorical variables

*BMI* body mass index, *EF* ejection fraction, *GP IIb/IIa* - glicoprotein IIb/IIIa, *LAD* left anterior descending artery, *LCx* left circumflex, *MI* myocardial infarction, *RCA* right coronary artery, *VF* ventricular fibrillation, *VT* ventricular tachycardia, *ACE* angiotensin-converting enzyme, *ARB* angiotensin receptor blocker, *ARNI* angiotensin receptor-neprilysin inhibitor, *MRAs* mineralocorticoid receptor antagonists, *PCI* percutaneous coronary intervention, *TIMI* thrombolysis in myocardial infarction, *SD* standard deviation.

The mean age was 53 ± 10 years for the hypothermia group and 58 ± 8 for controls. There were no significant differences between the groups in terms of age, sex, or body mass index (BMI). Dyslipidemia was more common in the group submitted to hypothermia [23 (79%) vs 4 (36%), p = 0.01]. There were no additional differences in the distribution of other cardiovascular risk factors such as hypertension, obesity, diabetes, family history of atherosclerosis, and current or previous smoking. There was also no difference between the groups regarding the myocardial wall affected by the infarction; the culprit artery; history of previous PCI; GRACE score at admission; association with dorsal wall myocardial infarction; troponin values (at admission, 8 h, 16 h, 24 h, and 48 h post-MI); time of chest pain; pain-to-balloon time; and door-to-balloon time. Additional characteristics are listed in Table 1.

### 3.2 CMR morphofunctional parameters and LGE (myocardial fibrosis)

Table 2 summarizes CMR morphofunctional parameters. Patients in the hypothermia group showed similar heart rates, LVEF, LVEDV, LV mass, LV mass indexed by BMI, LGE mass, IS divided by myocardial wall, LGE percentage, and salvage area, at both 5 and 30 days. There was no difference in final IS (26.4 ± 13.3% vs 31 ± 19.7%, p = 0.4), AAR (36.16 ± 14.31 vs 35.99 ± 16.81, p = 0.97), or mean final LVEF (40.4 ± 10.4% vs 46.2 ± 10.7%, p = 0.13) at 30 days. The presence of microvascular obstruction by CMR at 5 days was not significantly different between groups (19 (91%) vs 23 (79%) p = 0.65, for control vs hypothermia groups, respectively).

**Table 2:** CMR morphofunctional parameters.

	Control (n = 11)	Hypothermia (n = 29)	p-value
5 days			
Heart rate (bpm)	67.45±10.87 (60.1–74.7)	75.41±14.86 (69.7–81)	0.11
LVEF (%)	42.71±11.59 (34.9–50.5)	36.61±10.41 (32.6–40.5)	0.12
LVEDV (mL)	151.39±41.93 (123.2–179.5)	158.36±43.59 (141.7–174.9)	0.65
LV mass (g)	148.09±37.36 (122.9–173.1)	130.48±32.85 (117.9–142.9)	0.15
LV mass indexed by BMI	74.11±16.44 (63–85.1)	69.23±16.70 (62.8–75.5)	0.41
LGE mass (g)	43.53±27.35 (25.1–61.9)	36.74±19.77 (29.2–44.2)	0.39
LGE mass by culprit wall			
Anterior wall MI	48.9±39.11 (−48.27 to 146.07)	40.15±18.41 (29.52–50.79)	0.54
Inferior wall MI	41.52±24.79 (20.79–62.25)	33.56±21.08 (21.88–45.24)	0.42
LGE %	25.92±11.47 (18.2–33.6)	23.89±10.47 (19.9–27.8)	0.60
Salvage area (g)	23.94±14.23 (14.3–33.5)	25.79±15.56 (19.8–31.7)	0.73
Area at risk	36.16±14.31 (26.54–45.77)	35.99±16.81 (29.47–42.51)	0.97
30 days			
Heart rate (bpm)	59.09±8.84 (53.1–65)	64.62±13.31 (59.5–69.6)	0.21
LVEF (%)	46.2±10.73 (38.9–53.4)	40.41±10.44 (36.4–44.3)	0.13
LVEDV (mL)	167.391±46.8 (135.9–198.8)	159.11±41.72 (143.2–174.9)	0.59
LV mass (g)	126. 20±28.91 (106.7–145.6)	115.92±36.50 (102–129.8)	0.41
LV mass indexed by BMI	63.44±12.67 (54.9–71.9)	61.73±19.26 (54.4–69)	0.79
LGE mass (g)	31±19.77 (16.8–45.1)	26.44±13.35 (21.3–31.5)	0.42
LGE mass by culprit wall			
Anterior wall MI	27.24±21.41 (−25.96 to 80.44)	29.50±13.71 (21.58–37.42)	0.81
Inferior wall MI	32.66±20.58 (13.57–51.67)	23.58±12.79 (16.49–30.66)	0.21
LGE %	21.48±9.96 (14.3–28.6)	20.41±9.13 (16.9–23.8)	0.76
Salvage area (g)	46.23±13.45 (36.6–55.8)	45.07±14.03 (39.7–50.4)	0.82

Data are expressed as the mean ± SD for numeric variables and as absolute values and percentages for categorical variables, followed by 95% confidence intervals. p-values were not significantly different from 5–30 days in any parameters

*LV* left ventricle, *LVEF* left ventricle ejection fraction, *LVEDV* left ventricle end-diastolic volume, *LGE* late gadolinium enhancement, *CMR* cardiovascular magnetic resonance, *MI* myocardial infarction, *BMI* body mass index, *SD* standard deviation

### 3.3 Strain analysis

#### 3.3.1 Global strain

Table 3 provides an overview of the strain parameter analysis. No significant differences were observed in global RS, LS, or CS values between the hypothermia and control groups at both 5 and 30 days. Specifically, RS values at 5 days were similar between groups across different myocardial regions: infarcted (11.2 ± 16 vs 11.4 ± 14, p = 0.78), adjacent (22.4 ± 16.5 vs 24.5 ± 21.5, p = 0.09), and remote (28 ± 18 vs 28.62 ± 18.6, p = 0.91). CS values were likewise comparable at 5 days in all regions: infarcted (−5.4 ± 11.1 vs −6.5 ± 12.5, p = 0.09), adjacent (−13 ± 9 vs −13.1 ± 10.2, p = 0.93), and remote (−15.2 ± 10.4 vs −15.5 ± 10.7, p = 0.82).

**Table 3:** Strain parameters.

	Control (n = 11)	Hypothermia (n = 29)	p-value
5 days			
Global radial strain	18.5±4.9 (15.2–21.8)	16.38±6.53 (13.9–18.8)	0.34
Global longitudinal strain	−10.33±2.84 (−12.2 to −8.4)	−10.29±3.14 (−11.4 to −9)	0.97
Global circumferential strain	−12.22±2.78 (−14 to −10.3)	−10.98±3.36 (−12.2 to −9.7)	0.28
Radial strain per segment			
Infarcted area	11.46±14.02 (10.2–12.6)	11.23±16^a^ (10.4–12)	0.76
Adjacent area	24.58±21.56 (21.9–27.1)	22.43±16.53 (21.1–23.7)	0.11
Remote area	28.62±18.6^b^ (27.1–30)	28±18.06 (27.1–28.8)	0.45
Circumferential strain per segment			
Infarcted area	−6.51±10.6 (−7.4 to −5.6)	−5.4±11.18^c^ (−5.98 to −4.83)	0.04
Adjacent area	−13.12±10.36 (−14.3 to −11.8)	−13.05±9 (−13.7 to −12.3)	0.92
Remote area	−15.52±10.77^d^ (−16.3 to −14.6)	−15.2±10.46 (−15.7 to −14.7)	0.51
30 days			
Global radial strain	22.55±6.07 (18.4–26.6)	19.82±8.66 (16.5–23.11)	0.34
Global longitudinal strain	−12.48±2.79 (−14.3 to −10.6)	−12.37±3.14 (−13.5 to −11.1)	0.92
Global circumferential strain	−14.12±3.03 (−16.1 to −12)	−12.85±3.95 (−14.3 to −11.3)	0.34
Radial strain per segment			
Infarcted area	13.11±16.84 (11.6–14.5)	14.8±15.25^a^ (13.9–15.6)	0.03
Adjacent area	25.92±16.07 (23.7–28)	22.16±16.78 (20.7–23.5)	0.001
Remote area	31.79±18.54^b^ (30.3–33.2)	29.09±20.09 (28.1–30)	0.001
Circumferential strain per segment			
Infarcted area	−6.48±12.52 (−7.5 to −5.3)	−8±11.16^c^ (−8.6 to −7.3)	0.01
Adjacent area	−14.98±8.63 (−16.1 to −13.8)	−12.38±10.88 (−13.2 to −11.4)	0.001
Remote area	−17.16±9^d^ (−17.8 to −16.4)	−15.39±10.61 (−15.8 to −14.9)	0.001

Data are expressed as the mean ± SD for numeric variables and as absolute values and percentages for categorical variables, followed by 95% confidence intervals

SD *standard deviation*

a p = 0.001 (5 vs 30 days)

b p = 0.001 (5 vs 30 days)

c p = 0.001 (5 vs 30 days)

d p = 0.001 (5 vs 30 days)

#### 3.3.2 Remote areas

In remote segments of the control group, both RS (28 ± 18 to 31.7 ± 18.5, p = 0.001) and CS (−15.5 ± 10.7 to −17.1 ± 9, p = 0.001) significantly improved from 5–30 days. In contrast, no significant change was seen in the hypothermia group for RS (28.6 ± 18.6 to 29 ± 20, p = 0.09) or CS (−15.2 ± 10.4 to −15.3 ± 10.6, p = 0.61). At 30 days, remote area strain values were significantly better in the control group than in the hypothermia group for both RS (31.7 ± 18.5 vs 29 ± 20, p = 0.001) and CS (−17.1 ± 9 vs −15.3 ± 10.6, p = 0.001).

#### 3.3.3 Adjacent areas

RS did not improve in adjacent areas for either group (hypothermia group: 22.4 ± 16.5 to 22.1 ± 16.7, p = 0.80; control group: 24.5 ± 21.5 to 25.9 ± 16, p = 0.28). However, RS was significantly higher in the control group than in the hypothermia group at 30 days (25.9 ± 16 vs 22.1 ± 16.7, p = 0.01). CS values remained stable in the adjacent area for the hypothermia group (−13 ± 9 to −12.3 ± 10.8, p = 0.57), whereas the control group showed improvement (−13.1 ± 10.2 to −14.9 ± 8.6, p = 0.04), with greater contractility at 30 days compared to the hypothermia group (−12.3 ± 10.8 vs −14.9 ± 8.6, p = 0.001).

#### 3.3.4 Infarcted areas

**Fig. 3:**
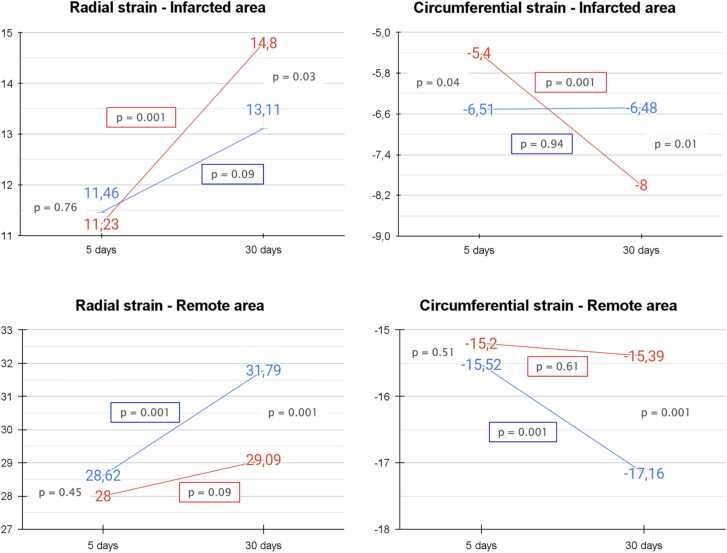
Radial and circumferential strain parameters at 5 and 30 days in infarcted and remote myocardial segment. This figure presents radial and circumferential strain changes over time in myocardial segments categorized as infarcted or remote. Red lines represent the hypothermia group, while blue lines denote the control group. At 30 days, infarcted segments in the hypothermia group show a notable increase in both radial and circumferential strain, suggesting enhanced contractility recovery compared to the control group. In contrast, remote segments in the control group demonstrate greater compensatory strain improvements, likely in response to reduced contractility within infarcted areas.

In infarcted segments, the hypothermia group showed significant recovery from 5–30 days in both RS (11.2 ± 16 to 14.8 ± 15.2, p = 0.001) and CS (−5.4 ± 11.1 to −8 ± 11.1, p = 0.001). By contrast, the control group exhibited no significant improvement (RS: 11.4 ± 14 to 13.1 ± 16.8, p = 0.09; CS: −6.5 ± 10.6 to −6.4 ± 12.5, p = 0.94). At 30 days, strain values in infarcted areas were significantly better in the hypothermia group for both RS (14.8 ± 15.2 vs 13.1 ± 16.8, p = 0.03) and CS (−8 ± 11.1 vs −6.4 ± 12.5, p = 0.01). Fig. 3 displays RS and CS parameters in infarcted and remote areas for both groups.

#### 3.3.5 Transmural infarcted areas (>50% LGE)

Transmural infarcted segments in the hypothermia group showed significant improvement in both RS (11.8 ± 13.2 to 8.17 ± 14.7, p = 0.001) and CS (−6.1 ± 10.9 to −3.1 ± 11.3, p = 0.001). No improvement was observed in the control group (RS: 8.9 ± 16.3 to 7.9 ± 11.8, p = 0.36; CS: −3.3 ± 12.9 to −4.3 ± 9.4, p = 0.22). At 30 days, the hypothermia group showed higher RS and CS values in transmural infarcted areas than the control group (RS: 11.8 ± 13.2 vs 8.9 ± 16.3, p = 0.001; CS: −6.1 ± 10.9 vs −3.3 ± 12.9, p = 0.001).

#### 3.3.6 Subendocardial infarcted areas (<50% LGE)

**Fig. 4:**
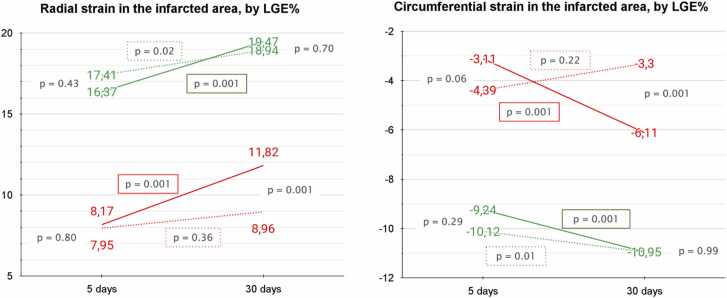
Radial and circumferential strain parameters at 5 and 30 days, categorized by the extent of transmurality in STEMI infarcted segments. This figure illustrates the evolution of radial and circumferential strain across infarcted myocardial segments with differing transmurality levels, measured at 5 and 30 days post-STEMI. Red lines represent transmural infarcted segments (>50% wall thickness), while green lines denote subendocardial infarcted segments (<50% wall thickness). Solid lines correspond to the hypothermia-treated group, indicating the effects of endovascular therapeutic hypothermia, and dashed lines represent the control group. Notably, transmural segments in the hypothermia group show a marked improvement in strain values at 30 days, highlighting a potential beneficial effect of the intervention on myocardial contractility in severely affected areas. *STEMI* ST-elevation myocardial infarction, *LGE* late gadolinium enhancement.

Both groups exhibited significant RS and CS improvement in subendocardial infarcted areas (hypothermia group RS: 19.4 ± 16.9 to 16.3 ± 16.7, p = 0.001; control group RS: 18.9 ± 15.8 to 17.4 ± 15.4, p = 0.02; hypothermia group CS: −10.9 ± 10.3 to −9.2 ± 9.7, p = 0.001; control group CS: −10.9 ± 10.8 to −10.1 ± 11.4, p = 0.01). However, no significant difference was observed between groups at 30 days (RS: 19.4 ± 16.9 vs 18.9 ± 15.8, p = 0.70; CS: −10.9 ± 10.3 vs −10.9 ± 10.8, p = 0.99). Fig. 4 illustrates RS and CS values at 5 and 30 days by STEMI transmurality level.

### 3.4 Intra and interobserver reproducibility

Good intraobserver and interobserver variabilities for the tissue-tracking CMR analysis of LS, CS, and RS were obtained, with all values higher than 0.85. The intraobserver ICCs (95% CI) for the measurement of LV LS, CS, and RS at 5 days were 0.97 (95% CI 0.88–0.99, p < 0.001), 0.92 (95% CI 0.62–0.98, p < 0.001), and 0.97 (95% CI 0.86–0.99, p < 0.001), respectively. Furthermore, the intraobserver ICCs (95% CI) for the measurement of LV LS, CS, and RS at 30 days were 0.96 (95% CI 0.81–0.99, p < 0.001), 0.96 (95% CI 0.82–0.99, p < 0.001), and 0.94 (95% CI 0.73–0.98, p < 0.001), respectively. The interobserver ICCs (95% CI) for the measurement of LV LS, CS, and RS at 5 days were 0.89 (95% CI 0.47–0.97, p < 0.005), 0.86 (95% CI 0.37–0.97, p < 0.009), and 0.93 (95% CI 0.66–0.98, p < 0.001), respectively. Furthermore, the interobserver ICCs (95% CI) for the measurement of LV LS, CS, and RS at 30 days were 0.97 (95% CI 0.87–0.99, p < 0.001), 0.92 (95% CI 0.60–0.98, p < 0.002), and 0.91 (95% CI 0.58–0.98, p < 0.003), respectively. Supplemental Table S1 contains additional information on reproducibility.

## 4 Discussion

We demonstrated for the first time that ETH in patients with STEMI submitted to PCI leads to a significant improvement in RS and CS in infarcted areas, resulting in greater contractility than infarcted areas in the control group. In contrast, there was a significant improvement in RS and CS in remote areas of the control group, resulting in greater contractility in these areas than in the group submitted to hypothermia.

Our results should be interpreted in the context of prior trials that evaluated hypothermia in STEMI patients who underwent primary PCI. Our findings are in accordance with previously published data regarding the noninfluence of ETH on LVEF and on the reduction of the infarct area [7], [8], [9], [10], [11], [12]. In our study, no statistically significant differences in IS were observed in either the control (25.92% ± 11.47% vs 21.48% ± 9.96%) or hypothermia (23.89% ± 10.47% vs 20.41% ± 9.13%) groups. No randomized clinical trial has ever shown a reduction in IS with ETH. However, a post hoc analysis suggested benefit in anterior STEMI patients who reached a target temperature <35°C before PCI [30], [31].

We found that infarcted areas in patients from the hypothermia group had greater improvements in RS and CS, regardless of the transmural extension of the LGE area. However, we also found that the contractility of transmural infarcted areas (LGE >50%) was greater than that in the control group at 30 days. In this scenario, there seems to be a possible protective effect of cooling on cardiomyocyte death in areas of ischemia. There are several mechanisms proposed for hypothermia-induced cardioprotection, but most of them have not yet been proven in humans [32], [33], [34], [35], [36], [37], [38].

In addition to these findings, our study provides a nuanced comparison to previous research on ETH as an adjunct to primary PCI in STEMI patients. The combined RAPID MI-ICE and CHILL-MI trials by Erlinge et al. [30] demonstrated the safety and feasibility of ETH but did not establish significant infarct size reduction, aligning with our results. Similarly, studies by Dixon et al. [7] and O'Neill et al. [8] reported mixed results for ETH’s impact on LVEF and infarct size. The CHILL-MI trial (2014) indicated potential benefits primarily in patients with shorter symptom onset, while Nichol et al. [10] and Noc et al. [11] found ETH did not consistently reduce infarct size or enhance MS across all groups. Our findings, in line with the COOL-MI InCor trial (2021), suggest that while ETH may not reduce overall infarct size, it significantly improves regional contractility in infarcted areas over a 30-day period, highlighting ETH's potential to support targeted myocardial recovery. These improvements in RS and CS underscore a distinct benefit of ETH in regional contractility and early remodeling, warranting further investigation to assess if these functional gains translate to long-term clinical outcomes.

Some of our study results serve as potential hypothesis generators. Residual myocardial viability within infarcted areas and myocardial compensatory contractility recruitment in remote segments might be involved in the pathophysiology of early LV remodeling after STEMI. The beneficial effect of endovascular hypothermia in infarcted areas appears to lead to a different need for compensation in remote myocardium.

Our results showed lower contractility in the infarcted area of the control group. This could have an effect of increasing the compensatory contractility mechanism in the remote area of the control group, in accordance with the pathophysiology of LV remodeling after MI [32]. In other words, the lower the contractility within the infarcted area, the higher the recruitment of contractility in the remote myocardium. Alternatively, the hypothermia group had a greater contractility recovery in the infarcted area; consequently, contractility increased less in the remote segments between 5 and 30 days, showing less myocardial contractility recruitment. More studies are needed to elucidate this phenomenon. A pooled analysis from the RAPID MI-ICE and CHILL-MI trials showed similar tendencies. In these studies, exploratory analysis revealed that patients with large infarcts and shorter symptom onset of reperfusion times benefited most from hypothermia [29].

## 5 Limitations

Our study has limitations. The study is retrospective and was conducted at a single center, with a small number of patients. No sample size calculation was performed for this specific study, as the sample size was a convenience sample based on the available cases from the original COOL-MI InCor trial database. However, experimental studies have shown that a sample size of 20 is able to detect an LV strain difference of 5% using CMR-FT technique (with 80% power and α error of 0.05) [35].

As the team responsible for conducting the endovascular therapeurtic hypothermia is an expert on the subject, there is a considerable risk of type II statistical error. The patients were not at high risk, as patients in Killip classes II–IV and those with cardiac arrest were excluded. Another limiting point is that we did not have CMR exams performed on the same day that hypothermia and PCI were performed, so we had to consider those obtained 5 days after the procedure as “acute phase” values. In addition, the last CMR was performed 30 days after the STEMI, so we cannot evaluate the long-term permanence of ETH impacts. Our results provide a better pathophysiological understanding of hypothermia effects in infarcted human myocardium. This might be clinically relevant for improved utilization of hypothermia advanced technology and with potentially better clinical results.

LGE can be influenced by edema and inflammation, which can resolve over time, making it a complex marker in the acute MI setting. Our study primarily used LGE to define infarct size and location. The concept of myocardial viability in the acute phase had been debated for a while and settled that LGE does reflect viability even in the acute phase. The area of acute LGE does not become viable over time, but the infarct area remodel and scar over time, changing its shape. Thus, the enhanced myocardial tissue in the acute phase is in fact not viable. We believe that myocardial strain offers an important functional assessment in this phase and serves as a valuable adjunct to tissue characterization [36].

## 6 Conclusions

In patients with anterior or inferior STEMI submitted to PCI, ETH increased RS and CS of the infarcted area, even in transmural infarcted areas. These findings might further contribute to a better understanding of the pathophysiology of early LV remodeling and ultimately suggest potential clinical value, which needs confirmation in larger studies.

## Funding

L.M.Q. received a scholarship from the National Council of Scientific and Technological Development (Conselho Nacional de Desenvolvimento Científico e Tecnológico – CNPq / Process number 161750/2021-4). J.C.N. is the recipient of a scholarship from the National Council of Scientific and Technological Development (Conselho Nacional de Desenvolvimento Científico e Tecnológico – CNPq / Process number 303448/2021-0). C.E.R. is the recipient of a scholarship from the National Council of Scientific and Technological Development (Conselho Nacional de Desenvolvimento Científico e Tecnológico – CNPq / Process number 309028/2021-3). R.A.F. is the recipient of a scholarship from Zerbini Foundation (Fundação Zerbini)/ Process number 001/2021.

## Author contributions

**Lucas de Mello Queiroz:** Writing – review & editing, Writing – original draft, Project administration, Methodology, Investigation, Formal analysis, Data curation, Conceptualization. **Luis Augusto Palma Dallan:** Methodology, Conceptualization. **Rafael Almeida Fonseca:** Methodology, Investigation, Formal analysis, Data curation, Conceptualization. **Ludhmila Abrahao Hajjar:** Methodology, Conceptualization. **Thatiane Facholi Polastri:** Methodology, Conceptualization. **Roberto Kalil Filho:** Methodology, Conceptualization. **Jose Carlos Nicolau:** Methodology, Conceptualization. **Sergio Timerman:** Methodology, Conceptualization. **Karl B. Kern:** Methodology, Conceptualization. **Carlos E. Rochitte:** Writing – review & editing, Writing – original draft, Supervision, Software, Project administration, Methodology, Investigation, Formal analysis, Data curation, Conceptualization.

## Ethics approval and consent

The present study was approved by the research ethics committee of the Hospital das Clínicas of the Faculty of Medicine of the University of São Paulo (no 4.535.256).

## Consent for publication

Not applicable.

## Declaration of competing interests

Lucas de Mello Queiroz and Jose Carlos Nicolau report financial support was provided by the National Council for Scientific and Technological Development. Jose Carlos Nicolau reports a relationship with Amgen Inc. that includes funding grants. Jose Carlos Nicolau reports a relationship with AstraZeneca that includes funding grants. Jose Carlos Nicolau reports a relationship with Bayer Corporation that includes funding grants. Jose Carlos Nicolau reports a relationship with CSL Behring that includes funding grants. Jose Carlos Nicolau reports a relationship with Daiichi Sankyo Inc. that includes funding grants. Jose Carlos Nicolau reports a relationship with DalCor Pharmaceuticals Canada Inc. that includes funding grants. Jose Carlos Nicolau reports a relationship with Esperion Therapeutics Inc. that includes funding grants. Jose Carlos Nicolau reports a relationship with Janssen Pharmaceuticals Inc. that includes funding grants. Jose Carlos Nicolau reports a relationship with Novartis that includes funding grants. Jose Carlos Nicolau reports a relationship with Novo Nordisk that includes funding grants. Jose Carlos Nicolau reports a relationship with Sanofi that includes funding grants. Jose Carlos Nicolau reports a relationship with Vifor Pharma Switzerland SA that includes funding grants. The other authors declare that they have no known competing financial interests or personal relationships that could have appeared to influence the work reported in this paper.
