# Supplementary material for: Hypothermia as an adjunctive therapy to percutaneous intervention after ST-elevation myocardial infarction—Effects on regional myocardial contractility
Source: J Cardiovasc Magn Reson. 2025 Feb 13;27(1):101850. doi: 10.1016/j.jocmr.2025.101850 (PMC11986222; doi:10.1016/j.jocmr.2025.101850)
Supplement: Supplementary file 1 — Supplementary material. [file mmc1.docx]

**SUPPLEMENTAL MATERIAL**

Table S1

**Supplemental Table 1** – Analysis of reproducibility at 5 and 30 days.

| **Variable** | | **ICC** | **95% CI** | **p-value** |  | | | **ICC** | **95% CI** | **p-value** |
| --- | --- | --- | --- | --- | --- | --- | --- | --- | --- | --- |
| **Interobserver** | | **5 days** | | | | | **30 days** | | | |
| LVEF | | 0.89 | 0.13 - 0.98 | <0.001 |  | | | 0.89 | 0.42 - 0.97 | <0.006 |
| LVEDV | | 0.86 | 0.30 - 0.97 | <0.003 |  | | | 0.97 | 0.88 - 0.99 | <0.001 |
| LGE % | | 0.93 | 0.68 - 0.98 | <0.001 |  | | | 0.98 | 0.93 - 0.99 | <0.001 |
| Edema % | | 0.95 | 0.76 - 0.99 | <0.001 |  | | | -- | -- | -- |
| Global RS | | 0.93 | 0.66 - 0.98 | <0.001 |  | | | 0.91 | 0.58 - 0.98 | <0.003 |
| Global CS | | 0.86 | 0.37 - 0.97 | <0.009 |  | | | 0.92 | 0.60 - 0.98 | <0.002 |
| Global LS | | 0.89 | 0.47 - 0.97 | <0.005 |  | | | 0.97 | 0.87 - 0.99 | <0.001 |
| **Intraobserver** | **5 days** | | | | | **30 days** | | | | |
| LVEF | | 0.99 | 0.97 - 0.99 | <0.001 |  | | | 0.98 | 0.88 - 0.99 | <0.001 |
| LVEDV | | 0.97 | 0.52 - 0.99 | <0.001 |  | | | 0.89 | 0.50 - 0.97 | <0.005 |
| LGE % | | 0.99 | 0.98 - 0.99 | <0.001 |  | | | 0.99 | 0.96 - 0.99 | <0.001 |
| Edema % | | 0.99 | 0.98 - 0.99 | <0.001 |  | | | -- | -- | -- |
| Global RS | | 0.97 | 0.86 - 0.99 | <0.001 |  | | | 0.94 | 0.73 - 0.98 | <0.001 |
| Global CS | | 0.92 | 0.62 - 0.98 | <0.001 |  | | | 0.96 | 0.82 - 0.99 | <0.001 |
| Global LS | | 0.97 | 0.88 - 0.99 | <0.001 |  | | | 0.96 | 0.81 - 0.99 | <0.001 |

ICC: Intraclass correlation coefficient. 95% CI: Confidence interval. All ICC values were >0.85.
